# Supplementary material for: Longer Brace Duration Is Associated with Lower Stress Levels and Better Quality of Life in Adolescents with Idiopathic Scoliosis
Source: Children (Basel). 2023 Jun 28;10(7):1120. doi: 10.3390/children10071120 (PMC10378022; doi:10.3390/children10071120)
Supplement: Supplementary file 1 [file children-10-01120-s001.zip › children-2321731-supplementary.pdf]

BSSQ

1. Pensi che l'apparenza del tuo corpo sia influenzata dal corsetto.
○ Assolutamente d’accordo
○ D’accordo
○ In disaccordo
○ Assolutamente in disaccordo

2. Mi pesa mostrarmi in pubblico indossando il corsetto.
○ Assolutamente d’accordo
○ D’accordo
○ In disaccordo
○ Assolutamente in disaccordo

3. Trovo spiacevoli le situazioni in cui gli altri possono vedere il mio corsetto.
○ Assolutamente d’accordo
○ D’accordo
○ In disaccordo
○ Assolutamente in disaccordo

4. Non mi interessa mostrare il corsetto.
○ Assolutamente d’accordo
○ D’accordo
○ In disaccordo
○ Assolutamente in disaccordo

5. Evito il contatto fisico in modo che le altre persone non si rendano conto che sto indossando il corsetto.
○ Assolutamente d’accordo
○ D’accordo
○ In disaccordo
○ Assolutamente in disaccordo

6. Quando scelgo i miei vestiti o porto i capelli lunghi, cerco di nascondere il corsetto.
○ Assolutamente d’accordo
○ D’accordo
○ In disaccordo
○ Assolutamente in disaccordo

7. Non mi importa mostrare il corsetto alle persone vicine (genitori, amici, compagni di scuola).
○ Assolutamente d’accordo
○ D’accordo
○ In disaccordo
○ Assolutamente in disaccordo

8. A causa del corsetto rinuncio alle attività ricreative e agli hobby che mi piacciono.
○ Assolutamente d’accordo
○ D’accordo
○ In disaccordo
○ Assolutamente in disaccordo
